# Coupling of nematic in-plane orientational ordering and equilibrium shapes of closed flexible nematic shells

**DOI:** 10.1038/s41598-023-37664-2

**Published:** 2023-07-01

**Authors:** Luka Mesarec, Wojciech Góźdź, Veronika Kralj-Iglič, Samo Kralj, Aleš Iglič

**Affiliations:** 1 Laboratory of Physics, Faculty of Electrical Engineering, University of Ljubljana, Tržaška Cesta 25, 1000 Ljubljana, Slovenia; 2 Institute of Physical Chemistry, Polish Academy of Sciences, Kasprzaka 44/52, 01-224 Warsaw, Poland; 3 Laboratory of Clinical Biophysics, Faculty of Health Sciences, University of Ljubljana, Zdravstvena 5, 1000 Ljubljana, Slovenia; 4 Department of Physics, Faculty of Natural Sciences and Mathematics, University of Maribor, Koroška Cesta 160, 2000 Maribor, Slovenia; 5 Condensed Matter Physics Department, Jožef Stefan Institute, Jamova 39, 1000 Ljubljana, Slovenia

**Keywords:** Biological physics, Surfaces, interfaces and thin films, Topological defects

## Abstract

The impact of the intrinsic curvature of in-plane orientationally ordered curved flexible nematic molecules attached to closed 3D flexible shells was studied numerically. A Helfrich-Landau-de Gennes-type mesoscopic approach was adopted where the flexible shell’s curvature field and in-plane nematic field are coupled and concomitantly determined in the process of free energy minimisation. We demonstrate that this coupling has the potential to generate a rich diversity of qualitatively new shapes of closed 3D nematic shells and the corresponding specific in-plane orientational ordering textures, which strongly depend on the shell’s volume-to-surface area ratio, so far not predicted in mesoscopic-type numerical studies of 3D shapes of closed flexible nematic shells.

## Introduction

It is of strong interest to identify and master generic mechanisms that drive 3D changes in geometrical shapes of diverse condensed matter systems^1–10^. Particularly intriguing are mechanisms which have the potential to generate a rich pallet of qualitatively different configurations, could be sensitively tuned, and exhibit universal features applicable to various physical systems. Different morphologies in general provide different functionalities, and such properties are, among other things, essential for complex systems to adapt to changes in their environments.

Of particular recent interest are mechanisms triggering 3D shape changes of soft matter objects, such as biological membranes^2,4–6,11–17^, elastomers^18^ or liquid crystalline (LC) bodies^7^. For instance, by varying the volume-to-surface area ratio, erythrocytes and liposomes transform among qualitatively different shape classes^2,19–22^. LC drops and shells^23,24^ could exhibit morphological changes by varying the surface tension strength and LC elasticity forces^7,8^. Via this mechanism, LC bodies could exhibit either a spherical, undulated, “flower”-like or filamentous structure. Furthermore, in elastomers one could trigger shape changes mechanically or remotely by appropriate external fields^18^.

Convenient model systems to study 3D shape changing mechanisms are closed 2D flexible shells^25,26^ exhibiting an in-plane structural order^27,28^ embedded in a 3D Euclidian space. There exist diverse experimentally accessible effectively thin flexible 2D ordered systems, for instance biological membranes or LC shells^23,24^. Furthermore, due to the relative simplicity of 2D flexible shells embedded in 3D space, several well established mathematical approaches modelling their behavior are available.

Topological defects (TDs)^29,30^ are inevitably formed in closed flexible shells exhibiting an in-plane order for nontoroidal topologies. They correspond to localized field distortions and are characterized by the winding number *m*^31^, which is a conserved value. Commonly, TDs carrying positive and negative values of *m* are referred to as defects and antidefects, respectively. According to the Gauss-Bonnet and Poincare-Hopf theorems^32–34^ the total winding number within a closed flexible shell equals 2 for spherical topology.

In our study we consider 3D shapes of closed 2D flexible thin shells with attached curved nematic rod-like molecules exhibiting a nematic orientational order (flexible nematic shells). We use a modified Landau-de Gennes-Helfrich model, where we introduce an additional term, which couples the curvature of flexible rod-like nematic molecules with the shells’s curvature field. We show that this term could generate a rich pallet of qualitatively different 3D shapes of flexible nematic shells with attached curved nematic molecules. The crucial geometric parameter of the 3D closed shapes of these nematic shells with volume *V* and surface area *A* is their relative/reduced volume $v=V/V_0$, where $V_0=4 \pi R^3/3$ represents the volume of a spherical shape with surface area *A* and radius $R=\sqrt{A/4 \pi }$. In numerical simulations of equilibrium shapes and the corresponding in-plane orientational ordering textures of closed flexible nematic shells, we impose a constraint on the value *v*. In previous related studies on the effect of orientational ordering of membrane attached curvature-inducing nematogens on nematic vesicle 3D shapes^11,35^, the relative volume *v* was not fixed in simulations. By contrast, in our study the value of *v* plays a key role in the determination of different 3D flexible shell shape classes.

## Results

We study the interplay between equilibrium shapes and the in-plane nematic orientational ordering of closed axially symmetric flexible shells with attached curved rod-like nematic molecules. The local geometry and order at the mesoscopic level of such nematic shells are described by the curvature field and nematic order parameter field of the nematic shell. Our model could describe diverse 2D curved manifolds exhibiting an in-plane nematic-type orientational order. Prototypical examples are nematic LC shells^23,24^ and biological membranes^3,4,6,10,11,13,14^.

The local curvature of the flexible nematic shell’s surface is described by the curvature tensor

1 $$\begin{aligned} \textbf{C}=C_{1}\textbf{e}_{1}\otimes \textbf{e}_{1}+C_{2}\textbf{e} _{2}\otimes \textbf{e}_{2}, \end{aligned}$$

where the unit eigenvectors $\left\{ \textbf{e}_{1},\textbf{e}_{2}\right\}$ determine the directions of maximal and minimal curvature and their eigenvalues $\left\{ C_{1},C_{2}\right\}$ are the corresponding two principal curvatures.

Orientational ordering on the surface is described by the surface order tensor $\textbf{Q}$, which can be expressed in the diagonal form as^36,37^

2 $$\begin{aligned} \textbf{Q}=\lambda (\textbf{n}\otimes \textbf{n}-\textbf{n}_{\perp }\otimes \textbf{n}_{\perp }), \end{aligned}$$

where $\left\{ \textbf{n},\textbf{n}_{\perp }\right\}$ are the eigenvectors of $\textbf{Q}$ corresponding to the eigenvalues of $\left\{ \lambda ,-\lambda \right\}$ and $\lambda$ is bound to be in an interval $[0,1/2]$. The lower bound ($\lambda =0$) represents the isotropic state without orientational order, while the upper bound ($\lambda =1/2$) represents the state with the maximal degree of orientational order. The orientation field on the flexible nematic shell surface is described by $\textbf{n}$ (nematic director field). Topological defects are signalled by $\lambda =0$.

In our model it is assumed that curved nematic molecules are homogeneously distributed throughout the flexible shell surface, but they can locally change their in-plane orientation. The total free energy functional of such curved nematic surface $\zeta$ is expressed as an integral of the sum of the flexible shell isotropic local bending energy density ($f_{\mathrm{b}}$), orientational condensation contribution ($f_{\mathrm{c}}$), intrinsic elastic (direct interaction) free energy density ($f_{\mathrm{i}}$) and the deviatoric bending energy density of curved nematic molecules ($f_{\mathrm{e}}$):

3 $$\begin{aligned} f_{\mathrm{b}}= & {} \frac{\kappa }{2}(C_{1}+C_{2}-C_{\mathrm{0}})^{2}, \end{aligned}$$

4 $$\begin{aligned} f_{\mathrm{c}}= & {} -\alpha ~Tr\textbf{Q}^{2}+\frac{\beta }{2}\left( Tr\textbf{ Q}^{2}\right) ^{2}, \end{aligned}$$

5 $$\begin{aligned} f_{\mathrm{i}}= & {} \frac{k_{\mathrm{i}}}{2}\left| \nabla _{\mathrm{s}} \textbf{Q}\right| ^{2}, \end{aligned}$$

6 $$\begin{aligned} f_{\mathrm{e}}= & {} \frac{k_\mathrm{e}}{2} (C-C_p)^2. \end{aligned}$$

The isotropic local bending energy density of the shell (Eq. 3) is described within the classical Helfrich’s spontaneous curvature model^1,14,21,38–40^. This term aims to minimise the isotropic bending energy of the flexible shell, where $\kappa$ is the shell local bending rigidity and $C_{\mathrm{0}}$ its isotropic spontaneous curvature.

The condensation term (Eq. 4) enforces nematic orientational ordering below the phase transition temperature $T_{\mathrm{c}}$. The equilibrium nematic ordering amplitude is given as $\lambda _{\mathrm{0}}=\sqrt{\alpha /\beta }$, where $\alpha =(T_{\mathrm{c}}-T)\alpha _{\mathrm{0}}$, $\alpha _{\mathrm{0}}$ and $\beta$ stand for positive Landau expansion material dependent coefficients and *T* is the temperature^36,37^.

The intrinsic elastic (direct interaction) term (Eq. 5) is weighted by the positive elastic constant $k_{\mathrm{i}}$^22,36,37,41^. This term enforces a spatially homogeneous nematic order and is locally minimised when neighbouring curved nematic molecules are parallel to each other. In this study the nematic order parameter correlation length $\xi = \sqrt{k_{\mathrm{i}} / |\alpha |}$ is the essential characteristic material dependent length.

An important property of our model is the introduction of the deviatoric bending energy of surface attached curved nematic molecules (Eq. 6), which describes their interaction with the shell’s curvature field at the mesoscopic level. In this term $C_p$ is the intrinsic (spontaneous) curvature of curved flexible nematic molecules. Note that the same term was originally introduced in^42,43^ to study the influence of curved rod-like proteins (e.g. BAR proteins^44^) attached to biological membranes, where direct interactions between proteins were not taken into account. In Eq. (6), the positive elastic constant $k_\mathrm{e}$ measures the coupling strength between curved nematic molecules and the flexible shell, and *C* is the local surface curvature seen by molecules. The curvature *C* can be expressed by the Euler relation:

7 $$\begin{aligned} C=H+D\cos {(2\eta )}, \end{aligned}$$

where $\eta$ is the angle between the normal plane of the first principal curvature $C_{1}$ and the normal plane in which the molecule is lying^45^. Furthermore, $D=(C_{1}-C_{2})/2$ is the curvature deviator and $H=(C_{1}+C_{2})/2$ the mean curvature at the given location on the surface^3,4,14,42,46^.

### Simulation results and discussion

In this section we present numerical results designed to study the interplay between the equilibrium 3D shapes and in-plane orientational ordering of closed thin and flexible nematic shells for different values of the relative volume. All lengths in the paper are scaled to $R=\sqrt{A/4\pi }$, which is the radius of a spherical shape with the same surface area as the surface area (*A*) of the investigated shell. We study the 3D shapes of closed 2D axisymmetric surfaces (flexible thin nematic shells) exhibiting a spherical topology.

**Figure 1:**
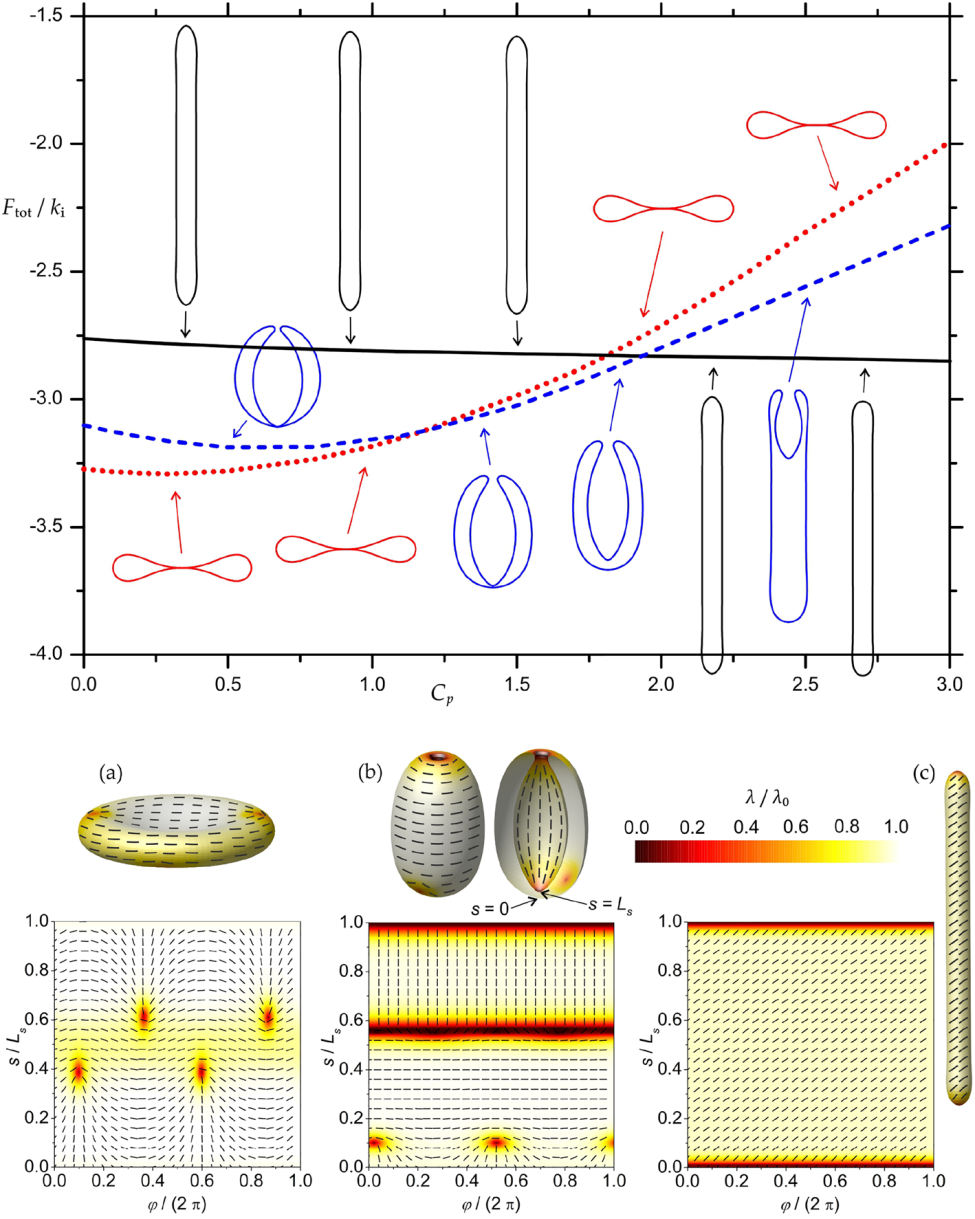
Sequence of equilibrium shape profiles of three different classes of nematic shell shapes calculated for different values of intrinsic curvature $C_p$ of attached curved rod-like nematic molecules. Since all lengths in our model are scaled to $R=\sqrt{A/4\pi }$, $C_p$ is dimensionless. The corresponding values of the total free energy $F_{\mathrm{tot}}$ are also given for each class of shapes (oblates, prolates, cup-shaped stomatocytes). Oblates are shown in red, stomatocytes in blue and prolates in black. In the bottom part of the figure, 3D visualisations and orientational ordering profiles of curved nematic molecules are given for each class of shapes, i.e. typical oblate (**a**), stomatocyte (**b**) and prolate (**c**). The degree of orientational order $\lambda /\lambda _0$ is denoted by the color code, while the orientation of curved nematic molecules is presented by the rods on each shape and in the ($\varphi ,s$)-plane. Here $\varphi$ is the azimuthal angle of the axisymmetric surface and *s* the arc length of the profile curve characterising the axisymmetric surface (see methods). Shapes were calculated for: $v=0.40$, $k_{\mathrm{e}}=k_{\mathrm{i}}/5$, $\kappa =k_{\mathrm{i}}/60$, $C_0=0$, $R/\xi =10$.

First we describe the influence of the intrinsic curvature of nematic molecules $C_p$ (see Eq. 6) on equilibrium 3D shapes of closed flexible nematic shells (Fig. 1). Note that $C_p$ is dimensionless since all lengths in our model are scaled to *R*. We predicted three qualitatively different classes of shapes (oblate, invaginated and prolate shapes) for different $C_p$ intervals. Each of these shape classes hosts a qualitatively different orientational ordering configuration and distribution of TDs, which is presented in the bottom part of Fig. 1. In experiments, the intrinsic curvature of anisotropic LC molecules can be altered via a UV-driven trans-cis transformation of LC isomers^47^; therefore, the sequence in Fig. 1 gives us an insight into the possible stable shapes and orientational ordering configurations of closed flexible nematic shells that are expected to be observed experimentally.

If we minimised only the free energy density of nematic shells associated with the energy of nematic curved rod-like molecules (Eq. 6), the prolate shapes (black shapes in Fig. 1) would be energetically most favourable for all $C_p$ values lower or equal to $1/r_{t}$, i.e. for the whole sequence presented in Fig. 1. Here, $r_{t}$ denotes the normalized radius of the prolate tube, which is determined by the value of the relative volume *v*^48^ (see Supplementary information). Curved nematic molecules can change the shape of prolates almost into perfect cylinders while adjusting their orientation angle to perfectly fit on the surface as shown in Fig. 1c. For the value of the relative volume $v=0.40$, which was used in the simulations presented in Fig. 1, prolates are quite narrow and consequently more curved. The high surface curvature on prolate tubes is penalized by the bending energy density (Eq. 3) and by the orientational condensation contribution (Eq. 4). Therefore, oblate shapes, which have on average a lower surface curvature, are energetically most favourable for low $C_p$ values in the interval $0 \le C_p \le 1.1$ (marked by the color red in Fig. 1). Note that curved nematic molecules with a low intrinsic curvature $C_p$ favour an oblate surface because the majority of the surface is relatively flat. The typical orientational ordering configuration on an oblate shape is presented in Fig. 1a. It hosts four $m=1/2$ TDs positioned just outside the equatorial region. Similar oblate/discocyte configurations were predicted before^22^, where discocytes were stable in a wide region for non-curved nematic molecules. In that study, the so-called extrinsic term^49,50^ was used, which has a similar effect as our deviatoric energy term (Eq. 6) by setting $C_p=0$.

When the intrinsic curvature of curved nematic molecules $C_p$ is increased, the oblate shape transforms into an invaginated stomatocyte shape. Stomatocytes are stable in the interval $1.2 \le C_p \le 1.9$ (marked blue in Fig. 1). Note that these stomatocytes are much more prolate than typical^51,52^, more spherical stomatocytes. They are shaped by the curved nematic molecules. The outer stomatocyte surface resembles a tube. Curved nematic molecules are oriented perpendicular to that tube (see Fig. 1b) in order to fit onto the surface curvature, which minimises the energy term given by Eq. (6). It is clear from Fig. 1 (see the blue shapes) that as $C_p$ increases, the radius of the outer stomatocyte tube-like surface decreases. The invaginated part of the stomatocyte surface has both principal curvatures $C_1$ and $C_2$ negative. Therefore, the curved nematic molecules with positive $C_p$ are frustrated there at any orientation. However, since the principal curvature along the meridians ($C_1$) has a lower absolute value than the principal curvature along the parallels ($C_2$), it is energetically favourable for the curved nematic molecules with positive $C_p$ to orient themselves along the meridians and in this way minimise the energy given by Eq. (6).

By further increasing the value of $C_p$, the outer stomatocyte tube-like surface gets thinner and longer, while the invaginated part of the surface is reduced in size because the relative volume of the shape *v* is kept constant. Thus the stomatocyte is gradually transformed into a prolate shape (blue shapes in Fig. 1). A typical stable stomatocyte from the sequence in Fig. 1 hosts two $m=1/2$ TDs on the outer surface and one $m=1$ TD on the invaginated surface (see Fig. 1b). The highly curved stomatocyte neck is topologically neutral, but it is highly disordered in a very narrow transient region where the curved nematic molecules drastically change their average orientation by 90 degrees (see Fig. 1b). Zooming in on the orientational ordering profile of the disordered narrow region of the neck, one could observe two $m=1/2$ defects and two $m=-1/2$ antidefects (Fig. 1b).

When the intrinsic curvature of curved nematic molecules $C_p \ge 2.0$, the prolate tubular shell shapes become the energetically most favourable shapes (marked black in Fig. 1). Highly curved nematic molecules are likely to induce prolate tubular shapes since they cannot perfectly fit on less curved surfaces, such as the surfaces of oblates and stomatocytes (see Fig. 1c). The calculated sequence of tubular shapes remains almost the same for all $C_p$ values because these shapes are mainly determined by the constraint of fixed relative volume^48^. If the intrinsic curvature of curved nematic molecules $C_p$ is lower or equal to the curvature of the prolate tubular shell ($C_p \le 1/r_{t}$), the curved nematic molecules can always adjust their orientation to perfectly fit onto the surface so that the energy term given by Eq. (6) is minimised. In Fig. 1c, a typical orientational ordering configuration shows that curved nematic molecules are oriented at a certain angle relative to the axis of the shape (they are tilted), which means that their curvature $C_p$ is still lower than the curvature of the prolate tubular shell $1/r_{t}$. The theoretically predicted spiral (tilted) organization of membrane proteins (Fig. 1c) was observed in many tubular membrane systems in vivo and in vitro, e.g. for F-BAR proteins^53,54^. A typical prolate shape, presented in Fig. 1c, exhibits one $m=1$ TD located at each pole.

**Figure 2:**
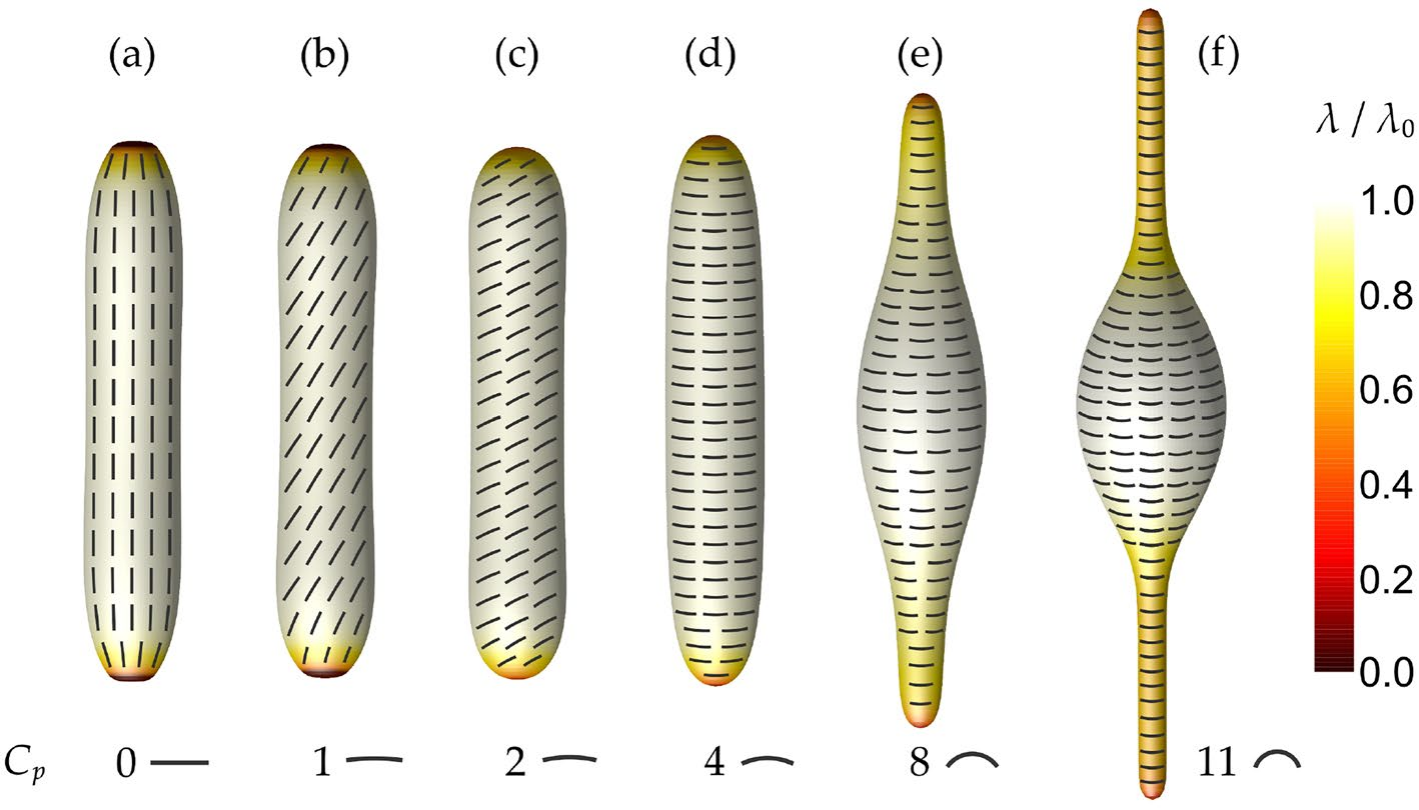
Equilibrium shapes and orientational ordering configurations on an increasing intrinsic curvature of curved nematic molecules $C_p$. Note that all lengths in our model are scaled to *R* so $C_p$ is dimensionless. The degree of orientational order $\lambda /\lambda _0$ is denoted by the color code, while the orientation of the curved nematic molecules is presented by differently curved rods on each shape. Shapes were calculated for: $v=0.60$, $k_{\mathrm{e}}=k_{\mathrm{i}}/5$, $\kappa =k_{\mathrm{i}}/60$, $C_0=0$, $R/\xi =10$.

Different orientation angles of curved nematic molecules attached to prolate shells and the effect of increasing the value of $C_p$ above the curvature of the prolate tube for a given relative volume^48^ are analyzed in Fig. 2. Since all lengths in our model are scaled to *R*, $C_p$ is dimensionless. Note that in the case presented in Fig. 2, prolate shapes represent equilibrium shapes in the whole $C_p$ sequence, while an oblate-stomatocyte-prolate transformation was observed in Fig. 1. This happened because the sequence in Fig. 2 was calculated at a higher relative volume ($v=0.60$) than the sequence in Fig. 1 ($v=0.40$). Therefore, the constraint for relative volume *v*, which allows more freedom (more excessive surface area) at lower values of *v*, plays a crucial role in determining equilibrium shapes. Oblate-stomatocyte-prolate shape transformations (Fig. 1) are possible only at lower values of *v*, while at higher values of *v* (Fig. 2) only prolates are stable. At higher values of *v*, prolate tubes are less narrow and less curved, which reduces the energy penalties by the bending energy density (Eq. 3) and by the orientational condensation contribution (Eq. 4), as already mentioned before. Furthermore, the free energy of curved nematic molecules (Eq. 6) on prolates is minimised as these molecules simply adjust their orientation to fit into the surface. Non-curved ($C_p=0$) nematic molecules are oriented parallel to the vertical axis of the prolate shape in Fig. 2a. In such a configuration, the energy term given by Eq. (6) is minimised on the majority of the surface. On each pole, we notice a flat membrane region (Fig. 2a) hosting a topological defect with the charge $m=1$. In this region, non-curved nematic molecules fit into the membrane well, but there is some bending energy (Eq. 3) penalty on the border of the region. If the bending energy effect was dominant, the shape would become more smoothly curved on both poles.

On increasing the value of $C_p$, nematic molecules start to tilt relative to the vertical symmetry axis in order to fit to the surface curvature and minimise the deviatoric bending energy term given by Eq. (6)—see Fig. 2b,c. In this process, shapes are not significantly altered, since, by adjusting their orientation, curved nematic molecules still perfectly fit into the majority of the surface and there is almost no additional energy penalty. The only significant shape changes in the sequence shown in Fig. 2a–c are observed at the poles. Curved nematic molecules change the surface shape at each pole in order to fit into the surface better and thus lower the energy penalty given by Eq. (6). In Fig. 2d, curved nematic molecules reach a perpendicular orientation relative to the vertical symmetry axis of the shape. From this point on, if the intrinsic curvature of curved nematic molecules $C_p$ is further increased, molecules cannot fit onto the tubular surface shown in Fig. 2a–d at any angle, which means that the elastic curvature energy of curved nematic molecules (Eq. 6) cannot be minimised only by adjusting their orientation as shown in the sequence in Fig. 2a–d. Therefore, in Fig. 2e,f, parts of the surface are squeezed by the curved nematic molecules to achieve a better fit in curvature and reduce the deviatoric bending energy penalty. An energetically perfect surface for highly curved nematic molecules would be a cylinder with the normalized radius defined as $r_{t}=1/C_p$. However, such a transformation would require a change of the relative volume *v*, which is constrained to the same value for all calculated shapes presented in Fig. 2. Consequently, the so-called $\phi$-shapes^55,56^ with two tubular protrusions are formed in Figs. 2e,f. The higher intrinsic curvature of curved nematic molecules $C_p$ leads to thinner tubular protrusions. In the calculations presented in Fig. 2, the curved nematic molecules are assumed to be homogeneously distributed throughout the whole surface. While highly curved nematic molecules fit perfectly on thin membrane tubular protrusions, they cannot fit on the less curved middle part of the shell (Figs. 2e,f) which results in the deviatoric bending energy penalty (Eq. 6) in that region. If the local density of the nematic molecules would be allowed to vary, the deviatoric bending energy (Eq. 6) could be further reduced by a migration of the curved nematic molecules from the middle inflated part (dictated by the constraint of constant relative volume *v*) to the highly curved protrusions^45^. If the molecules were not distributed over the entire surface, the costly TDs could also be avoided, which would reduce the energy penalty given by the energy terms described by Eqs. (4) and (5).

The formation of tubular (cylindrical) shapes due to rod-like proteins and different orientations of differently curved proteins on the tubes were predicted also in^11,35^, where the in-plane orientational ordering of curvature-inducing nematogens and their effect on the membrane structure was studied by Monte Carlo simulations on discretized triangulated surfaces of a spherical topology. The authors predicted that curved proteins would be oriented perpendicular to the tube (Fig. 2d in our case), while non-curved proteins (straight proteins) would be oriented along the tube axis (Fig. 2a in our case). To predict the spiral (tilted) orientation of proteins relative to the tube axis (Figs. 1c and 2b,c in our case), it was assumed^11,35^ that proteins are described by two intrinsic (spontaneous) curvatures as previously suggested in^3,4,42^. We show that the spiral organization of curved nematic molecules is possible also when the molecules are rod-like (Figs. 1c and 2b,c), i.e. described only by one intrinsic curvature ($C_p$ in our case).

**Figure 3:**
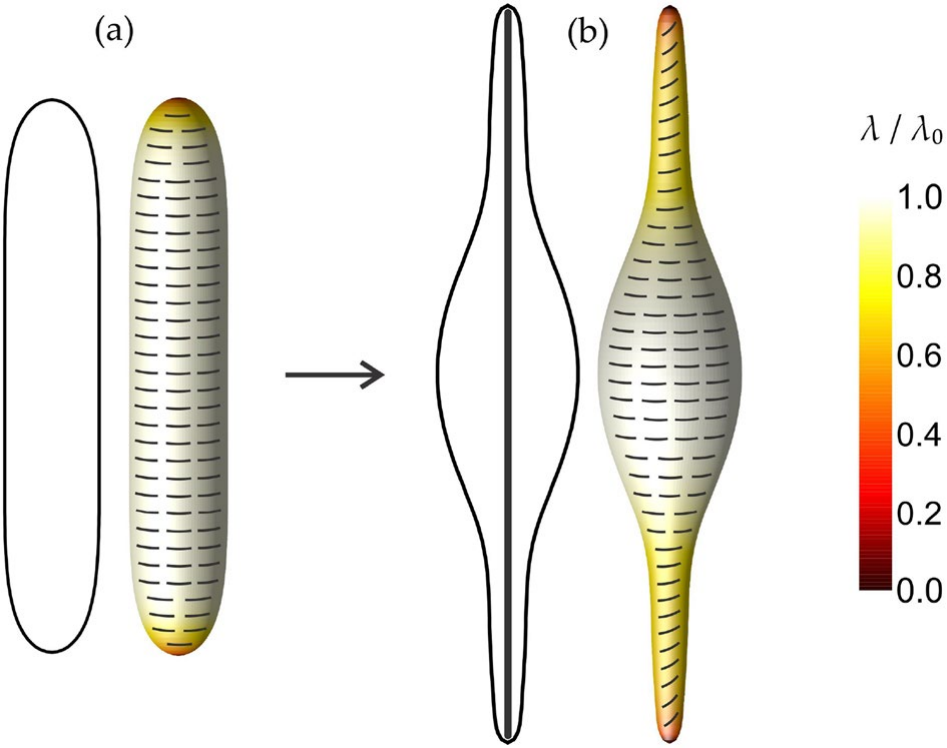
The influence of an external force on equilibrium shapes and orientational ordering configurations at a constant relative volume. The shape (**a**) is calculated in the absence of any external force, while the shape (**b**) is stretched by the rigid rod inside the shell. The degree of orientational order $\lambda /\lambda _0$ is in both cases denoted by the color code, while the orientations of curved nematic molecules are presented by the curved rods on each shape. Parameters: $v=0.60$, $k_{\mathrm{e}}=k_{\mathrm{i}}/5$, $C_p=5.0$, $\kappa =k_{\mathrm{i}}/15$, $C_0=0$, $R/\xi =10$.

The effect of an external force acting on a closed flexible nematic shell shape is presented in Fig. 3. Such a force could occur in biological membranes as a result of a growing actin cytoskeleton inside the vesicle^45^. In Fig. 3b the force is schematically shown as a rigid rod stretching the closed flexible shell. In numerical simulations, the force is modelled by setting a constraint on the minimal height of the shape. Fig. 3a shows the prolate shape of a closed flexible nematic shell with the curved nematic molecules oriented perpendicular to the axis, where the shape was calculated without considering the influence of an external stretching force. When the shape is stretched, two thinner tubular protrusions are formed on both sides (Fig. 3b) since the relative volume *v* of the shape is assumed to remain constant in the process. Curved nematic molecules with $C_p=5.0$, which fit onto the tube surface at a perpendicular orientation in the configuration presented in Fig. 3a, are not as curved as the curvature of thin tubular protrusions on the shape in Fig. 3b (note that all lengths in our model are scaled to $R=\sqrt{A/4\pi }$ so $C_p$ is dimensionless.) Consequently, curved nematic molecules adjust their orientation in these highly curved protrusion regions to fit into the surface—they are slightly tilted relative to the shape’s axis (Fig. 3b). A similar phenomenon was studied in^45^ for the case of closed membrane shapes containing curved BAR proteins, but without considering the direct interaction term (Eq. 5).

**Figure 4:**
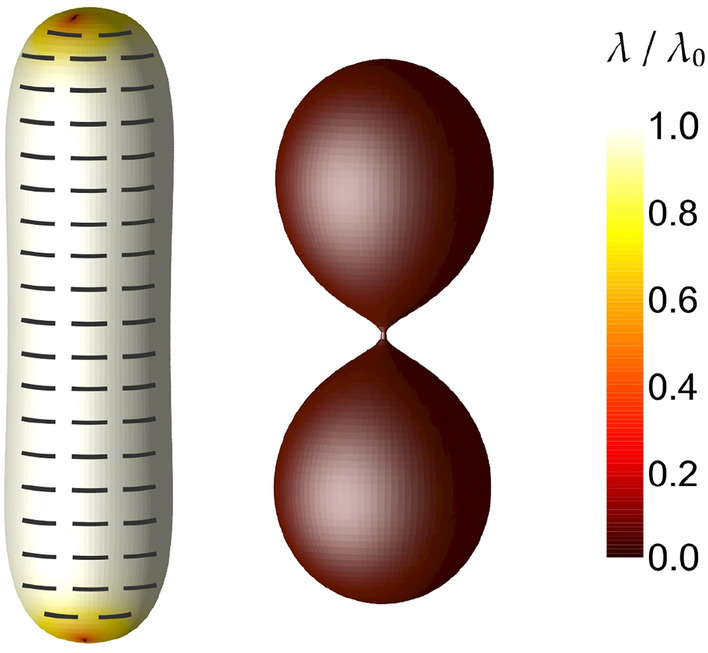
Shape change when moving from the ordered nematic (left-side shape) to the disordered isotropic (right-side shape) phase. The degree of orientational order $\lambda /\lambda _0$ is in both cases denoted by the color code. In the nematic phase, the orientations of curved nematic molecules are presented by the curved rods, while all orientations are equally probable in the isotropic phase. Parameters: $v=0.70$, $C_p=1.5$, $\kappa =0$, $k_{\mathrm{e}}=k_{\mathrm{i}}/5$, $R/\xi =10$. In the nematic (isotropic) phase we set $\alpha >0$ ($\alpha <0$).

Finally, we analyzed temperature-driven shape reconfigurations (Fig. 4). We compared two qualitatively different states; the nematic state below the phase transition temperature $T_c$ (left-side shape in Fig. 4) and the isotropic state above $T_c$ (right-side shape in Fig. 4). In simulations, we have quenched the system from a temperature corresponding to $T>T_c$ to $T<T_c$ (i.e., we changed the sign of $\alpha$, where $\alpha =(T_{\mathrm{c}}-T)\alpha _{\mathrm{0}}$). In the nematic phase ($\alpha >0$), we can analyze the degree of orientational order and the orientation of the curved nematic molecules on the surface. In the isotropic state ($\alpha <0$), there is no orientational order ($\lambda =0$), which is marked by the dark red color on the shape’s surface. We can observe that the temperature has a significant effect on equilibrium shape and the orientational ordering configuration. At this stage, we cannot claim that the phase transition temperature and the morphological transition coincide. Note that the nematic correlation length varies across the transition, which can lead to qualitatively different results when the temperature is gradually varied. Namely, at the phase transition temperature, the nematic correlation length diverges, and the energy penalty of the topological defects varies greatly with a change in temperature.

The shapes in Fig. 4 were calculated without the isotropic bending energy contribution ($\kappa =0$ in Eq. 3). They are therefore influenced only by curved nematic molecules, which are homogeneously distributed throughout the whole surface. In the nematic phase (below the phase transition temperature, $\alpha >0$), these curved nematic molecules possess an orientational order (left-side shape in Fig. 4). Except on the poles, curved nematic molecules are parallel to each other, which minimises the deviatoric bending energy of curved nematic molecules given by Eq. (5). On the poles, topological defects occur due to purely topological reasons. We can observe two defects with charges $m=1/2$ on each pole. The left-side shape in Fig. 4 is a cylinder with curved nematic molecules oriented perpendicular to the vertical axis of the shape.

The right-side shape in Fig. 4 was calculated in the disordered isotropic phase (above the phase transition temperature, $\alpha <0$), where the orientational ordering is lost throughout the surface, i.e. all orientations of curved nematic molecules are equally probable. This creates an isotropic spontaneous curvature preference, which is similar to the effect of the isotropic bending energy given by Eq. (3). In the case of randomly oriented non-curved nematic molecules ($C_p=0$), the (membrane) surface has the preference to be locally flat, which is the same as choosing $C_0=0$ in the isotropic bending term given by Eq. (3). If the surface is covered by randomly oriented curved nematic rod-like molecules ($C_p \ne 0$), then there is a tendency towards a local isotropic curvature, which is the same as choosing $C_0 \ne 0$ in Eq. (3). The latter effect is clearly demonstrated in Fig. 4, where can we observe an undulated (necklace-like) shape in the disordered isotropic phase (right-side shape) with the thin neck connecting different membrane compartments. Similar shapes are usually predicted within the spontaneous curvature model^1,2,20,21,38^ (Eq. 3) for $C_0 \ne 0$^51^.

## Conclusions

The coupling between the 3D equilibrium shapes of 2D closed shells and their 2D in-plane nematic orientational ordering was studied theoretically. We assumed strong adhesion of curved flexible nematic molecules on the flexible shell support. In addition to the free energy terms associated with in-plane orientationally ordered curved nematic rod-like molecules, we included in the total free energy functional also the isotropic bending energy of the flexible shell as supporting material for the attached curved nematic molecules (Eq. 3), which favours a locally flat surface and penalizes highly curved surface regions. The value of relative volume *v* played a key role in the determination of the equilibrium shapes of closed flexible nematic shells. In biological membranes, the Gibbs-Donnan equilibrium determines the osmotic equilibrium between two solutions separated by the membrane. Since the membrane is permeable only to certain molecules (ions) in both solutions, the relative volume *v* of the closed membrane (vesicle) is fixed for the given osmotic conditions in the system^57,58^.

At lower values of the relative volume *v*, we observed that the intrinsic curvature of curved nematic molecules $C_p$ has a drastic effect on the equilibrium 3D shapes of closed nematic shells and consequently also on the in-plane orientational ordering configurations of the shells. The oblate-stomatocyte-prolate sequence of closed nematic shells was predicted on increasing $C_p$ along with newly observed prolate-stomatocyte hybrid shapes (Fig. 1). In^45^ we speculated that an external stretching force is required to predict the tilted (spiral) orientation of curved nematic molecules. In the present study, such an in-plane orientational ordering configuration was theoretically predicted also for prolate shapes in the absence of an external stretching force (Fig. 2). Without considering the external stretching force, we also predicted so-called $\phi$-shapes with two tubular protrusions if the value of $C_p$ is increased above the curvature of the prolate tube (Fig. 2). The influence of an external stretching force on equilibrium shapes and orientational ordering configurations was presented in Fig. 3, where we observed a slightly tilted orientation of curved nematic molecules in highly curved protrusion regions.

Lastly, we have shown that temperature-driven shape reconfigurations when moving from ordered (nematic) to disordered (isotropic) phase can lead to the formation of undulated shapes with extremely thin disordered necks (Fig. 4) that are likely to rupture for example due to external mechanical or electrostatic forces^59^. For the right-side shape in Fig. 4 we assume that the temperature is high enough so that the deviatoric term (Eq. 6) cannot enforce orientational ordering anywhere on the surface (see Supplementary information^60^). Increasing the temperature above the phase transition temperature could therefore lead to the formation of thin necks and consequently to the fission of the shell into separate compartments.

The main focus of the work was to show the variety of qualitatively different configurations that can be stabilized simply by varying the intrinsic curvature of the molecule ($C_p$). Thus, our goal was to show that a rich palette of qualitatively different configurations can be achieved by changing a single molecular property, i.e., the intrinsic shape of the molecule. Note that the detailed critical conditions depend on several parameters. For example, varying specific membrane material properties or the characteristic linear dimension (i.e., *R* in our study) would lead to quantitatively different content shown in Fig. 1. Moreover, topological defects play an important role since they are energetically costly. Therefore, by manipulating their core structure and detailed placement, one can have a relatively strong quantitative impact on the stability diagrams. The TD core size is given approximately by the order parameter correlation length $\xi$. Note that $\xi$ diverges when approaching the orientational order-disorder phase transition at the phase transition temperature $T_c$. Near $T_c$, it holds $\xi \propto 1/\sqrt{|\alpha |}$, where $\alpha = \alpha _0 (T_c-T)$. Namely, in 2D the nematic-isotropic phase transition is continuous^61^. Therefore, we expect a significantly quantitatively different behavior of the system just by varying the temperature. Moreover, there are several indications (see^62^ and references therein) that the proximity of the phase transition temperature in biological membranes is evolutionarily beneficial. Therefore, significant changes in the configuration of the membrane energy landscape are expected by varying the temperature, and this is also the focus of our planned future study. In addition, the relative strength of intrinsic ($C_p$) and deviatoric (*D*) curvature may also strongly influence the placement of TDs and thus the stability diagram of competing membrane structures. Moreover, the character of the order-disorder phase transition might depend on the membrane thickness^61,62^. Namely, in 3D bulk system, the isotropic-nematic phase transition is discontinuous because the condensation free energy term (see Eq. 4) also contains a term proportional to $Tr\textbf{Q}^{3}$, which is allowed by the nematic (i.e., axial) symmetry, while this term vanishes in 2D^62^. Thus, just by varying the membrane thickness, the character of the order-disorder phase transition can vary. Finally, in systems of our interest nonequilibrium phenomena could play an important role. In summary, the quantitative details of our model system can be greatly varied by changing various model parameters and quantities.

## Methods

We conducted simulations on two-dimensional (2D) closed axisymmetric shells with spherical topology. These shells are assumed to have a surface of revolution with rotational symmetry about the *z*-axis within the Cartesian coordinate system (*x*, *y*, *z*), defined by the unit vectors ($\textbf{e}_{x}$, $\textbf{e}_{y}$, $\textbf{e}_{z}$). To construct such surfaces, the profile curve is rotated around the $\textbf{e}_{z}$ axis by an angle of $\varphi = 2 \pi$. A generic point lying on an axisymmetric surface is given by^22,41^:

8 $$\begin{aligned} \textbf{r}=\rho (s)\cos {\varphi }~\textbf{e}_{x}+\rho (s)\sin {\varphi }~\textbf{e} _{y}+z(s)~\textbf{e}_{z}, \end{aligned}$$

where $\rho (s)$ and *z*(*s*) are the coordinates of the profile curve in the $(\rho ,z)$-plane, $\varphi \in [0,2\pi ]$ stands for the azimuthal angle and *s* is the arc length of the profile curve. On a surface of revolution, the parallels and meridians are lines of principal curvature. We establish that the principal directions $\textbf{e}_{1}$ and $\textbf{e}_{2}$ (Eq. 1) align with the meridians (where $\varphi$ is constant) and parallels (where *s* is constant), respectively.

### Calculation of the profile curve

To determine the shapes of shells within our Helfrich-Landau-de Gennes-type mesoscopic approach, we introduce an angle $\theta (s)$, which represents the angle of the tangent to the profile curve with respect to the plane that is perpendicular to the axis of rotation $\textbf{e}_{z}$. The profile curve of an axisymmetric surface is calculated as^22,41,63–65^:

9 $$\begin{aligned} \rho (s)=\int _{0}^{s}\!\cos {\theta (s^{\prime })}\,\mathrm{d}s^{\prime },~ z(s)=\int _{0}^{s}\!\sin {\theta (s^{\prime })}\,\mathrm{d}s^{\prime }. \end{aligned}$$

For closed and smooth surfaces, the boundary conditions are as follows: $\theta (0)=0$, $\theta (L_{\mathrm{s}})=\pi$ and $\rho (0)=\rho (L_{\mathrm{s}})=0$, where $L_{\mathrm{s}}$ represents the length of the profile curve. Note that these are the only constraints on $\rho (s)$ and $\theta (s)$. Additionally, the angle function $\theta (s)$ is approximated using the Fourier series^22,41,63–65^:

10 $$\begin{aligned} \theta (s)=\theta _{\mathrm{0}}\frac{s}{L_{\mathrm{s}}}+\sum _{i=1}^{N}a_{\mathrm{ i}}\sin \left( \frac{\pi }{L_{\mathrm{s}}}i\cdot {s}\right) , \end{aligned}$$

where the Fourier series involves *N* Fourier modes ($N=80$ for the simulations presented in this paper), with $a_{\mathrm{i}}$ representing the Fourier amplitudes. The angle at the north pole of the axisymmetric surface is denoted as $\theta _{\mathrm{0}}$ and is equal to $\theta _{\mathrm{0}}=\theta (L_{\mathrm{s}})=\pi$. The local principal curvatures, $C_{1}$ and $C_{2}$, are calculated as $\frac{d\theta (s)}{ds}$ and $\frac{\sin (\theta (s))}{\rho (s)}$, respectively^22,41^.

### Calculation of nematic order

The parameterization for the nematic order tensor (defined in Eq. 2) is given by^22,36,37,41^:

11 $$\begin{aligned} \textbf{Q}=q_{\mathrm{0}}(\textbf{e}_{\mathrm{1}}\otimes \textbf{e}_{\textsf{ 1}}-\textbf{e}_{\mathrm{2}}\otimes \textbf{e}_{\mathrm{2}})+q_{\mathrm{m}}( \textbf{e}_{\mathrm{1}}\otimes \textbf{e}_{\mathrm{2}}+\textbf{e}_{\mathrm{2} }\otimes \textbf{e}_{\mathrm{1}}), \end{aligned}$$

where $q_{\mathrm{0}}$ and $q_{\mathrm{m}}$ are scalar functions. The angle $\eta$ between the normal plane of the first principal curvature $C_1$ and the normal plane in which the molecule is lying (see Eq. 7) can be determined from the following equations^37^:

12 $$\begin{aligned} \cos {(2\eta )}=\frac{q_{\mathrm{0}}}{\sqrt{q_{\mathrm{0}}^2+q_{\mathrm{m}}^2}}, ~~\sin {(2\eta )}=\frac{q_{\mathrm{m}}}{\sqrt{q_{\mathrm{0}}^2+q_{\mathrm{m}}^2}}. \end{aligned}$$

The standard functions used to represent the first fundamental form on axisymmetric surfaces in the $(\varphi ,s)$ coordinates are^22,36,41^:

13 $$\begin{aligned} E:=\textbf{r}_{\mathrm {,\varphi }}\cdot \textbf{r}_{\mathrm {,\varphi }}=\rho (s)^{2},~F:= \textbf{r}_{\mathrm {,\varphi }}\cdot \textbf{r}_{\mathrm{,s}}=0,~G:=\textbf{r}_{ \mathrm{,s}}\cdot \textbf{r}_{\mathrm{,s}}=\rho (s)_{\mathrm{,s}}^{2}+z(s)_{ \mathrm{,s}}^{2}, \end{aligned}$$

where a comma denotes the differentiation. The Jacobian determinant is:

14 $$\begin{aligned} J(s):=\sqrt{EG-F^{2}}=\rho (s)\sqrt{\rho (s)_{\mathrm{,s}}^{2}+z(s)_{\mathrm{ ,s}}^{2}}~. \end{aligned}$$

Since the meridians on a surface of revolution are also geodesics, their geodesic curvature is $\kappa _{\mathrm{g1}}=0$. On the other hand, the geodesic curvature of the parallels can be expressed as^22,36,41^:

15 $$\begin{aligned} \kappa _{\mathrm{g2}}=\frac{E_{\mathrm{,s}}}{2E\sqrt{G}}=\frac{\rho (s)_{ \mathrm{,s}}}{\rho (s)\sqrt{\rho (s)_{\mathrm{,s}}^{2}+z(s)_{\mathrm{,s}}^{2} }}. \end{aligned}$$

The Gaussian curvature and mean curvature of an axisymmetric surface can be obtained as^22,36,41^:

16 $$\begin{aligned} K(s)= & {} -\frac{z(s)_{\mathrm{,s}}(z(s)_{\mathrm{,s}}~\rho (s)_{\mathrm{,ss} }-z(s)_{\mathrm{,ss}}~\rho (s)_{\mathrm{,s}})}{\rho (s)\left( \rho (s)_{ \mathrm{,s}}^{2}+z(s)_{\mathrm{,s}}^{2}\right) ^{2}}, \end{aligned}$$

17 $$\begin{aligned} H(s)= & {} \frac{\rho (s)(z(s)_{\mathrm{,s}}~\rho (s)_{\mathrm{,ss}}-z(s)_{\mathrm{,ss}}~\rho (s)_{\mathrm{,s}})-z(s)_{\mathrm{,s}}(\rho (s)_{\mathrm{,s} }^{2}+z(s)_{\mathrm{,s}}^{2})}{\rho (s)\left( \rho (s)_{,s}^{2}+z(s)_{,s}^{2}\right) ^{3/2}}. \end{aligned}$$

The local principal curvatures, $C_{\mathrm{1}}$ and $C_{\mathrm{2}}$, are related to the Gaussian curvature (*K*) and mean curvature (*H*) through the following expressions:

18 $$\begin{aligned} K=C_{\mathrm{1}}C_{\mathrm{2}},~2H=C_{\mathrm{1}}+C_{\mathrm{2}}. \end{aligned}$$

The surface gradient of a scalar function $\psi$ in the $(\varphi ,s)$ coordinates on an axisymmetric surface can be expressed as^22,41^:

19 $$\begin{aligned} \nabla _{\mathrm{s}}\psi =\frac{1}{\sqrt{G}}\frac{\partial \psi }{\partial s} \textbf{e}_{\mathrm{1}}+\frac{1}{\rho (s)}\frac{\partial \psi }{\partial \varphi } \textbf{e}_{\mathrm{2}}, \end{aligned}$$

while the surface gradients of $\textbf{e}_{\mathrm{1}}$ and $\textbf{e}_{\mathrm{2}}$ are^37^:

20 $$\begin{aligned} \nabla _{\mathrm{s}}\textbf{e}_{\mathrm{1}}= & {} \kappa _{\mathrm{g1}}\textbf{e }_{\mathrm{2}}\mathbf {\otimes e}_{\mathrm{1}}+\kappa _{\mathrm{g2}}\textbf{e} _{\mathrm{2}}\mathbf {\otimes e}_{\mathrm{2}}-C_{\mathrm{1}}\mathbf {v\otimes e }_{\mathrm{1}}, \end{aligned}$$

21 $$\begin{aligned} \nabla _{\mathrm{s}}\textbf{e}_{\mathrm{2}}= & {} -\kappa _{\mathrm{g1}}\textbf{ e}_{\mathrm{1}}\mathbf {\otimes e}_{\mathrm{1}}-\kappa _{\mathrm{g2}}\textbf{e }_{\mathrm{1}}\mathbf {\otimes e}_{\mathrm{2}}-C_{\mathrm{2}}\mathbf {v\otimes e}_{\mathrm{2}}, \end{aligned}$$

where $\textbf{v}=\textbf{e}_{\mathrm{1}} \times \textbf{e}_{\mathrm{2}}$ is the surface normal. For a given closed shell geometry, the free energy density associated with nematic in-plane ordering is expressed in terms of fields $q_{\mathrm{0}}$ and $q_{\mathrm{m}}$.

### Numerical simulations

In numerical simulations, we obtain the equilibrium shapes of closed shells and their corresponding nematic ordering textures. The equilibrium nematic textures are computed using the standard Monte Carlo method, where the shell surface in the coordinates $(\varphi ,s)$ is represented as the network of $101\times 101$ points. In order to obtain the total free energy, the numerical integration is performed over the shell surface with the aid of the Jacobian determinant *J*(*s*) (Eq. 14). The equilibrium shell shapes are determined through the numerical minimisation of a function of multiple variables (the Fourier amplitudes $a_{\mathrm{i}}$ and $L_{\mathrm{s}}$)^63–65^. First, the Monte Carlo method is employed to obtain the equilibrium nematic configuration on a fixed shape. Subsequently, the surface shape is adjusted based on the current nematic texture, and this process is repeated iteratively until the equilibrium shell shape and nematic texture are obtained. In the minimisation process, we maintain a constant surface area (*A*) and volume (*V*) of the shell to establish a fixed value for the relative volume of the shell (*v*).

The parameterization for all the shapes is the same. The different calculated shapes are given by different sets of Fourier amplitudes $a_{\mathrm{i}}$ and $L_{\mathrm{s}}$. With the same parameterization during minimization of the free energy, it is possible to simulate the transition from the shape of higher energy to the shape of lower energy. It is also possible to obtain two different shapes with different energies for the same set of parameters. In special cases, we can obtain the same energies for two different shapes and the same set of parameters. Such points would mark the morphological transition based on the energy (see the transitions between different shapes in Fig. 1). The transitions between oblates, prolates and stomatocytes are discontinuous (1st order) because they have different geometries (the shapes don’t belong to the same class of shapes). In our simulations, we first determine the candidates for equilibrium shapes from different initial conditions and then determine the actual equilibrium shapes by comparing the energy values. This type of numerical procedure is especially important near the transition point, where the different shapes (shape classes) have similar energy values.

## Supplementary Information

Supplementary Information.

## Data Availability

All data generated or analysed during this study are included in this published article and its Supplementary information files.
