# Supplementary material for: Coupling of nematic in-plane orientational ordering and equilibrium shapes of closed flexible nematic shells
Source: Sci Rep. 2023 Jul 1;13:10663. doi: 10.1038/s41598-023-37664-2 (PMC10314910; doi:10.1038/s41598-023-37664-2)
Supplement: Supplementary file 1 — Supplementary Information. [file 41598_2023_37664_MOESM1_ESM.pdf]

# Coupling of nematic in-plane orientational ordering and equilibrium shapes of closed flexible nematic shells

Luka Mesarec[1*], Wojciech Góźdź[2], Veronika Kralj-Iglič[3], Samo Kralj[4,5] and Aleš Iglič[1]

[1*]Laboratory of Physics, Faculty of Electrical Engineering, University of Ljubljana, Tržaška cesta 25, Ljubljana, 1000, Slovenia.
[2]Institute of Physical Chemistry, Polish Academy of Sciences, Kasprzaka 44/52, Warsaw, 01-224, Poland.
[3]Laboratory of Clinical Biophysics, Faculty of Health Sciences, University of Ljubljana, Zdravstvena 5, Ljubljana, 1000, Slovenia.
[4]Department of Physics, Faculty of Natural Sciences and Mathematics, University of Maribor, Koroška cesta 160, Maribor, 2000, Slovenia.
[5]Condensed Matter Physics Department, Jožef Stefan Institute, Jamova 39, Ljubljana, 1000, Slovenia.

*Corresponding author. E-mail: luka.mesarec@fe.uni-lj.si;

# Supplementary information

## 1 Neck rupture

In Figure 5 we propose a possible topologically defect-driven neck rupture mechanism leading to vesiculation [1, 2]. In Fig. 5, equilibrium shapes and orientational ordering textures are calculated for non-curved nematic rod-like molecules ($C_p = 0$ in Eq. (6)). The values of the parameters are set such that the isotropic bending energy density (Eq. (3)) has a dominant role in the flexible shell shape determination. A prolate shape for $v = 0.80$ (Fig. 5a), an oblate shape for $v = 0.60$ (Fig. 5b) and a stomatocyte shape for

$v = 0.40$ (Fig. 5c) are predicted. These are typical values of relative volume $v$ of Helfrich's spontaneous curvature model for zero spontaneous curvature $C_0 = 0$ in Eq. (3) [3, 4], where stomatocytes are stable for $v < 0.59$, oblates for $0.59 \leq v \leq 0.65$ and prolates for $v > 0.65$. By analyzing the orientational ordering configurations, we observed that the introduced deviatoric term for the energy of curved nematic molecules (Eq. (6)) [5, 6] has, for $C_p = 0$, a very similar effect as the extrinsic term used in our previous studies [4, 7, 8]. The prolate shape in Fig. 5a hosts two $m = 1/2$ defects in the vicinity of each pole. On the majority of the prolate surface, the curved nematic molecules are oriented parallel to the axis of the shape in order to minimise the deviatoric energy given by Eq. (6). The oblate shape in Fig. 5b hosts four $m = 1/2$ defects, which are expelled from the highly deviatoric equatorial region because the energy term given by Eq. (6) enforces nematic molecules with $C_p = 0$ to be perpendicular to the symmetry axis of the closed nematic shell shape in that region.

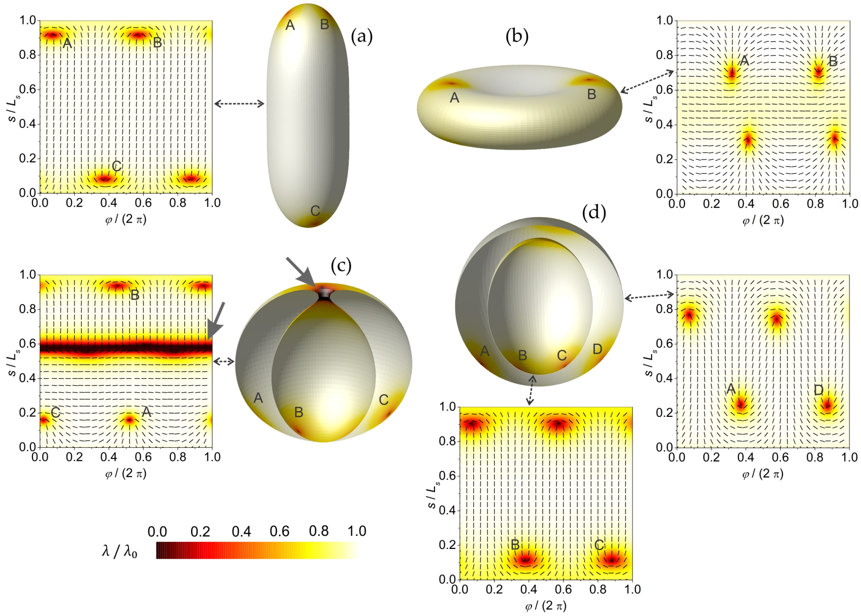


**Fig. 5** Dependence of equilibrium closed nematic shell shapes and orientational ordering textures of rod-like nematic molecules with zero intrinsic curvature $C_p = 0$ on decreasing relative volume ($v = 0.80$ in panel (a), $v = 0.60$ in panel (b), $v = 0.40$ in panel (c)) that leads to the formation of a stomatocyte with a thin neck (panel (c)). The rupture of a highly disordered stomatocyte neck region would lead to a fission process, which is presented in panel (d). The degree of orientational order $\lambda/\lambda_0$ is denoted by the color code on each shape and in the $(\varphi, s)$-plane. The orientation of rod-like nematic molecules is presented in the $(\varphi, s)$-plane. Topological defects are denoted by capital letters. Parameters: $k_e = k_i/5$, $C_p = 0$, $\kappa = k_i/15$, $C_0 = 0$, $R/\xi = 10$.

Fig. 5 also describes the effect of decreasing relative volume $v$ on the calculated equilibrium shapes and orientational ordering configurations. When $v < 0.59$, stomatocytes become the energetically most favorable shapes. On the stomatocyte surface (Fig. 5c), we can observe two $m = 1/2$ TDs on the outer surface and two $m = 1/2$ TDs on the invaginated part of the surface. Furthermore, we can observe a highly disordered neck region, which is denoted by arrows in Fig. 5c. In that region, the orientation of rod-like nematic molecules with $C_p = 0$ is subject to a sudden change because the orientation tendencies below and above the neck differ. Below the neck region (on the positively curved outer stomatocyte surface) nematic molecules are oriented perpendicular to the shape's vertical symmetry axis, while they are oriented parallel to the axis above the neck region (on the negatively curved invaginated stomatocyte surface). Note that the disordered neck region is not a topological defect, i.e. it has no topological charge. Nevertheless, like topological defects, it represents a source of large local elastic penalties as the ordering field in that region is essentially melted [9–11]. The local interactions between the neighbouring nematic molecules within the neck are therefore weakened, which might result in a neck rupture, leading to the fission process [1, 2, 12–14]. Consequently, two distinct closed shells are formed, where the smaller one resides inside the larger one as shown in Fig. 5d. The highly distorted neck region vanishes and two additional $m = 1/2$ TDs are created on each surface for topological reasons (the total topological charge of a closed surface with the topology of a sphere must be equal to 2). When only the direct interaction term (Eq. (5)) is taken into account, stomatocyte necks usually host two $m = -1/2$ topological antidefects [15]. In some cases, topological antidefects can be expelled from the center of the catenoid-like neck region as a consequence of strong orientational ordering enforced by the deviatoric term (Eq. (6)) in that region, which would cause TDs to move slightly above and below the neck center [16] (see also Section 5 of Supplementary information). The fission process as a consequence of antidefects accumulated in thin membrane necks was described in some of our previous works [1, 2, 12, 16] for undulated (necklace-like) shells.

## 2 Typical shapes in the isotropic phase

The typical shapes of membrane and lipid vesicles [17, 18], i.e. stomatocytes, discocytes and prolates, were predicted within Helfrich's spontaneous curvature model [14, 19]. Similar shapes can be predicted also within the theoretical model presented in this work for the disordered (isotropic) phase and a homogeneous distribution of non-curved ($C_p = 0$) nematic molecules (Fig. 6). The bending energy of the flexible supporting shell (Eq. (3)) is neglected in Fig. 6. Nematic molecules in the isotropic phase are highly disordered ($\lambda = 0$ on the whole surface, see Fig. 6), which means that all orientations of nematic molecules are equally probable. If all orientational directions of non-curved nematic molecules are equally probable, the best fit for them is the locally flat surface. This creates a similar effect as choosing a zero spontaneous curvature

in the Helfich spontaneous curvature model. Both models therefore favour a locally flat shell surface, which is the reason for the similarity between the calculated equilibrium shapes of stomatocytes (Fig. 6a), discocytes (Fig. 6b) and prolate shapes (Fig. 6c).

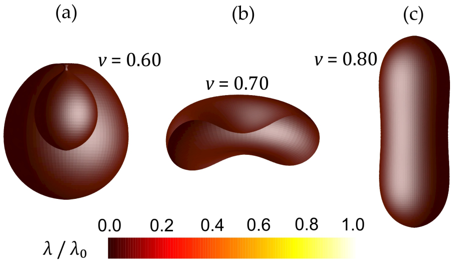


**Fig. 6** Typical stable shapes in the disordered isotropic phase ($\alpha < 0$) for non-curved ($C_p = 0$) nematic molecules, calculated in the absence of the bending energy term of the flexible supporting shell ($\kappa = 0$) for different relative volume values. The degree of orientational order $\lambda/\lambda_0$ is denoted by the color code on each shape. All orientations of straight rod-like nematic molecules are equally probable since the shells are in the isotropic phase. Other model parameters are: $k_\mathrm{e} = k_\mathrm{i}/5$, $R/\xi = 10$.

# 3 Temperature effect - whole sequence

We analyzed temperature-driven shape transformations and orientational ordering reconfigurations (Fig. 7). We compared two qualitatively different states; the nematic state below the phase transition temperature $T_c$ (left-side shape in each panel of Fig. 7) and the isotropic state above the phase transition temperature $T_c$ (right-side shape in each panel of Fig. 7). These two states are determined by the parameter $\alpha = (T_\mathrm{c} - T)\alpha_0$. In the nematic phase ($\alpha > 0$), we can analyze the degree of orientational order and the orientation of curved nematic molecules attached to the surface of the flexible shell. In the isotropic state ($\alpha < 0$), there is no orientational order ($\lambda = 0$), where we assume that the temperature is high enough so we could neglect the orientational ordering driven by the deviatoric energy term Eq. (6) (see Section 5 of Supplementary information and Ref. [20]), which is, however, not the best approximation for the thin necks of undulated shapes. In Fig. 7 the surface regions in the isotropic state are marked by the dark red color on the shapes' surfaces. We can observe that in all cases the temperature has a significant effect on equilibrium shapes and orientational ordering configurations.

Shapes in the upper row of Fig. 7 were calculated in the absence of the shell local bending energy contribution ($\kappa = 0$ in Eq. (3)). The shapes are in this case influenced only by the curved nematic molecules, which are assumed to be homogeneously distributed throughout the surface. In the nematic phase (below the phase transition temperature, $\alpha > 0$), these rods possess an orientational order (left-side shapes in each panel). Except on the poles, the curved

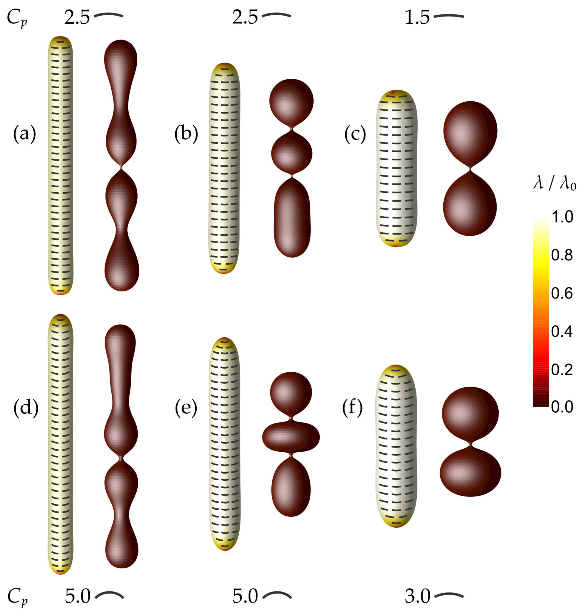


**Fig. 7** Shape changes when moving from the ordered nematic (left-side shape in each panel) to the disordered isotropic (right-side shape in each panel) phase. The degree of orientational order $\lambda/\lambda_0$ is denoted by the color code on each shape. In the nematic phase, the orientations of curved rod-like nematic molecules are presented by the curved rods, while all orientations are equally probable in the pure isotropic phase. Configurations in the upper row were calculated without the local bending energy contribution of the supporting shell ($\kappa = 0$), while the local bending energy of the shell (Eq. (3)) was considered only for the shapes in the lower row ($\kappa = k_\mathrm{i}/15$, $C_0 = 0$). For comparison, the relative volume values were set as follows: $v = 0.45$ for panels (a) and (d), $v = 0.55$ for panels (b) and (e), $v = 0.70$ for panels (c) and (f). Other model parameters are: $k_\mathrm{e} = k_\mathrm{i}/5$, $R/\xi = 10$. In the nematic (isotropic) phase we assumed that $\alpha > 0$ ($\alpha < 0$).

nematic molecules are parallel to each other, which minimizes the energy term given by Eq. (5). On the poles, topological defects occur. We predicted either one defect with charge $m = 1$ or two defects with charges $m = 1/2$ on each pole. The left-side shapes in the upper row of Fig. 7 are cylinders with curved nematic molecules oriented perpendicular to the vertical rotational symmetry axis of each shape. Results only slightly change when we take into account the bending energy of the supporting shell (($\kappa \neq 0$ in Eq. (3), which is presented in the lower row of Fig. 7. Shapes in the lower row are calculated for the same values of relative volume $v$ for comparison. In this case, the shape is determined by competition between the Helfrich local bending energy of the supporting shell (enforcing a locally flat surface for $C_0 = 0$) and the deviatoric bending energy of the curved nematic molecules (enforcing a locally curved surface that is the best fit for curved rod-like nematic molecules with the intrinsic curvature $C_p$). These two effects oppose each other, so a higher $C_p$ is required to obtain tubular shapes (left-side shapes in the lower row of Fig. 7).

The right-side shapes in Fig. 7 were calculated in the isotropic phase (above the phase transition temperature, $\alpha < 0$), where it is assumed that the orientational ordering is melted throughout the surface, including the necks, i.e. all orientations of curved nematic molecules are equally probable. This creates an isotropic spontaneous curvature preference, which is similar to the effect of the isotropic Helfrich local bending energy. Considering the orientational ordering due to the deviatoric energy term Eq. (6) also above the phase transition temperature would decrease the energy of the thin necks, but would not considerably change the shapes (see also [21] and Section 5 of this Supplementary information) in the disordered isotropic phase presented in Fig. 7. In the case of randomly oriented non-curved nematic molecules ($C_p = 0$), the (membrane) surface has the preference to be locally flat, which is the same as choosing a zero spontaneous curvature in the Helfrich local bending energy. If the surface is covered by randomly oriented curved nematic molecules ($C_p \neq 0$), then there is a tendency towards a local isotropic curvature, which is the same as if choosing $C_0 \neq 0$ in Eq. (3). The latter effect is clearly demonstrated in Fig. 7, where we can observe undulated (necklace-like) shapes in the isotropic phase (right-side shapes in each panel). Typical for these shapes are thin necks connecting the different membrane parts. Such shapes are typically obtained within the Helfrich spontaneous curvature model for zero spontaneous curvature [22, 23]. Note that in our case, the shapes in the upper row of Fig. 7 are calculated in the absence of the local bending energy of the supporting flexible shell ($\kappa = 0$ in Eq. (3)), while a zero spontaneous curvature of the supporting shell ($C_0 = 0$) is considered for the shapes in the lower row of Fig. 7. In the latter case, a higher $C_p$ is required to obtain the undulated (necklace-like) shapes because $C_0 = 0$ in Eq. (3) has an opposing effect. Furthermore, extremely thin necks are not energetically favorable when we consider a zero spontaneous curvature of the supporting shell and neglect the orientational ordering driven by the deviatoric bending energy (Eq. (6)) in thin necks. Consequently, necks in the lower row are slightly wider compared to those in the upper row.

# 4 Tubular shapes

We considered prolate tubular limiting shapes [18] with a half sphere on each pole (Fig. 8). We showed that the ratio between the length of cylinder $L$ and the radius of cylinder tube $R_t$ is determined by the value of the relative volume of closed shell $v$.

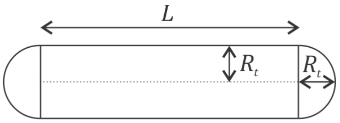


**Fig. 8** Schematic presentation of a tubular (cylindrical) shape with a half sphere on each pole. Here, $R_t$ is the radius and $L$ the length of the tube.

The volume of the shape presented in Fig. 8 is given by $V = 4\pi R_t^3/3 + \pi R_t^2 L$ and its surface area by $A = 4\pi R_t^2 + 2\pi R_t L$. The radius of a sphere with the same surface area $A$ can be calculated as $R = \sqrt{A/(4\pi)} = \sqrt{R_t^2 + R_t L/2}$, while its volume is given by $V_0 = 4\pi R^3/3 = 4\pi(R_t^2 + R_t L/2)^{3/2}/3$. The relative volume of the shape presented in Fig. 8 is thus given by:

$$v = \frac{V}{V_0} = \frac{\frac{4}{3}\pi R_t^3/ + \pi R_t^2 L}{\frac{4}{3}\pi(R_t^2 + \frac{R_t L}{2})^{3/2}} = \frac{1 + \frac{3L}{4R_t}}{(1 + \frac{L}{2R_t})^{3/2}}. \tag{S1}$$

The above equation can be solved to obtain the $L/R_t$ ratio for each value of the relative volume $v$. Therefore, the limiting tubular shape is determined by the relative volume constraint only.

The normalized intrinsic curvature of curved rod-like nematic molecules $\tilde{C}_p$ that would perfectly fit onto the limiting tubular surface presented in Fig. 8 at the orientation perpendicular to the tube axis is given by:

$$\tilde{C}_p = \frac{1}{r_t} = \frac{R}{R_t} = \frac{\sqrt{R_t^2 + R_t L/2}}{R_t} = \sqrt{1 + \frac{L}{2R_t}}, \tag{S2}$$

where $r_t = R_t/R$ is the normalized tube radius. Note that the ratio $L/R_t$ depends on the relative volume $v$ (Eq. (S1)). The ratio $L/R_t$ (Eq. (S1)) and $\tilde{C}_p$ (Eq. (S2)) are plotted in Fig. 9 for different values of relative volume $v$.

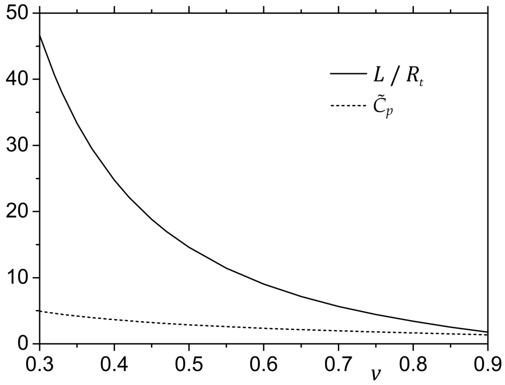


**Fig. 9** Dependence of the ratio $L/R_t$ (Eq. (S1)) and $\tilde{C}_p$ (Eq. (S2)) on relative volume $v$.

# 5 Average orientation of curved rod-like molecules

The deviatoric bending energy of a single curved rod-like molecule (Eqs. (6) and (7)) is a function of the orientation of molecule $\theta$. By inserting the Euler

relation (Eq. (7)) into the deviatoric bending energy of the curved rod-like molecule (Eq. (6)) we obtain:

$$f_{\mathrm{e}} = \frac{k_{\mathrm{e}}}{2}(H^2 + 2HD\cos(2\theta) - 2HC_p \\ -2DC_p\cos(2\theta) + D^2\cos^2(2\theta) + C_p^2). \tag{S3}$$

Focusing only on the orientation-dependent part of the energy, we can write:

$$f_{\mathrm{e}}(\theta) = -\gamma_1\cos(2\theta) + \gamma_2\cos^2(2\theta), \tag{S4}$$

where the functions $\gamma_1 = k_{\mathrm{e}}D(C_p - H)$ and $\gamma_2 = k_{\mathrm{e}}D^2/2$ include different constants and curvatures from Eq. (S3), which are determined by comparing Eq. (S3) and Eq. (S4). The average orientation of a single curved rod-like molecule can be calculated by using the methods of statistical physics [20, 24]:

$$\langle\cos(2\theta)\rangle = \frac{\int_0^{2\pi}\cos(2\theta)\exp(-f_{\mathrm{e}}(\theta)/k_BT)\mathrm{d}\theta}{\int_0^{2\pi}\exp(-f_{\mathrm{e}}(\theta)/k_BT)\mathrm{d}\theta}, \tag{S5}$$

where $k_B$ is the Boltzmann constant and $T$ the absolute temperature. The result of the numerical integration of the above equation for a special case of cylindrical geometry ($H = D$) is presented in Fig. 10, where the average orientation of the curved rod-like molecule is plotted as a function of $k_{\mathrm{e}}D^2/k_BT$.

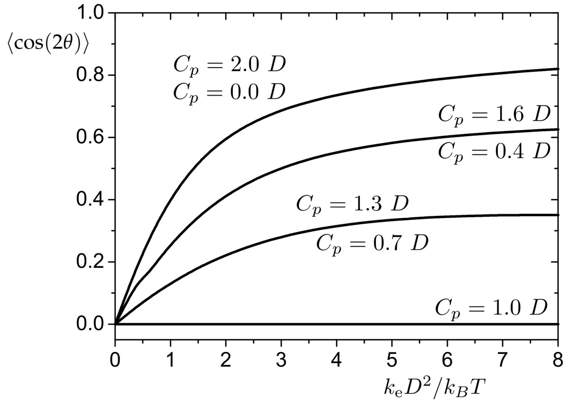


**Fig. 10** The average orientation $\langle\cos(2\theta)\rangle$ of a rod-like molecule as a function of $k_{\mathrm{e}}D^2/k_BT$ for $H = D$ (cylindrical geometry). $C_p$ values for different plots are given in the graph.

We can observe in Fig. 10 that the orientational ordering promoted by deviatoric bending energy (Eq. (6)) increases with increasing $k_{\mathrm{e}}D^2/k_BT$. Evidently, this term induces strong orientational ordering in highly curved anisotropic regions with high $D$. In our special case of cylindrical geometry, the strongest

ordering is induced for $C_p = 0.0$ (non-curved molecules oriented along the cylinder geometrical axis) and for $C_p = 2.0\ D$ (curved molecules that fit perfectly into the cylinder when oriented perpendicular to the cylinder axis). On the other hand, molecules with $C_p = 1.0\ D$ coincide with the cylinder curvature at the 45° angle leading to $\langle \cos(2\theta) \rangle = 0$ for all $k_{\rm e}D^2/k_BT$ values (quadrupolar orientation). Other $C_p$ values represent cases between these two extremes. It is also clear from Fig. 10 that orientational ordering is lost/melted in the limit of high temperatures $T$. In fact, we have competition between the anisotropic curvature enforced ordering increased with the increasing $k_{\rm e}D^2$ and random fluctuations that increase with increasing $T$. Therefore, even in systems with a relatively high temperature and no orientational ordering on the majority of the surface, highly curved anisotropic surface regions such as thin (cylindrical) membrane necks could still possess some degree of orientational order.

Note that changing the temperature impacts not only the deviatoric energy $f_{\rm e}$ but also other terms in the free energy (in particular $f_{\rm i}$ and $f_{\rm c}$). The intrinsic term $f_{\rm i}$ can only enforce parallel alignment of neighbouring molecules in the nematic phase where $T < T_c$ (see Eqs. (4) and (5)).

# References


[1] Jesenek, D., Perutková, Š., Góźdź, W., Kralj-Iglič, V., Iglič, A., Kralj, S.: Vesiculation of biological membrane driven by curvature induced frustrations in membrane orientational ordering. International Journal of Nanomedicine **8**(1), 677–687 (2013)

[2] Mesarec, L., Iglič, A., Kralj, S.: Topological defect driven membrane fission. Adv Biomed Res Innov **4**, 2 (2018)

[3] Mesarec, L., Fošnarič, M., Penič, S., Kralj Iglič, V., Kralj, S., Góźdź, W., Iglič, A.: Numerical study of membrane configurations. Advances in Condensed Matter Physics **2014** (2014)

[4] Mesarec, L., Góźdź, W., Iglič, A., Kralj-Iglič, V., Virga, E., Kralj, S.: Normal red blood cells' shape stabilized by membrane's in-plane ordering. Scientific Reports **9**(1), 1–11 (2019)

[5] Iglič, A., Slivnik, T., Kralj-Iglič, V.: Elastic properties of biological membranes influenced by attached proteins. Journal of Biomechanics **40**(11), 2492–2500 (2007)

[6] Perutková, Š., Kralj-Iglič, V., Frank, M., Iglič, A.: Mechanical stability of membrane nanotubular protrusions influenced by attachment of flexible rod-like proteins. Journal of Biomechanics **43**(8), 1612–1617 (2010)

[7] Napoli, G., Vergori, L.: Extrinsic curvature effects on nematic shells. Physical Review Letters **108**(20), 207803 (2012)

[8] Selinger, R.L.B., Konya, A., Travesset, A., Selinger, J.V.: Monte carlo studies of the xy model on two-dimensional curved surfaces. The Journal of Physical Chemistry B **115**(48), 13989–13993 (2011)

[9] Bowick, M., Nelson, D.R., Travesset, A.: Curvature-induced defect unbinding in toroidal geometries. Physical Review E **69**(4), 041102 (2004)

[10] Mermin, N.D.: The topological theory of defects in ordered media. Reviews of Modern Physics **51**(3), 591 (1979)

[11] Kurik, M.V., Lavrentovich, O.: Defects in liquid crystals: homotopy theory and experimental studies. Soviet Physics Uspekhi **31**(3), 196 (1988)

[12] Gongadze, E., Mesarec, L., Kralj, S., Kralj-Iglič, V., Iglič, A.: On the role of electrostatic repulsion in topological defect-driven membrane fission. Membranes **11**(11), 812 (2021)

[13] Kamien, R.D.: The geometry of soft materials: a primer. Reviews of Modern physics **74**(4), 953 (2002)

[14] Helfrich, W.: Elastic properties of lipid bilayers: theory and possible experiments. Zeitschrift für Naturforschung C **28**(11-12), 693–703 (1973)

[15] Mesarec, L., Kurioz, P., Iglič, A., Góźdź, W., Kralj, S.: Curvature-controlled topological defects. Crystals **7**(6), 153 (2017)

[16] Kurioz, P., Mesarec, L., Iglič, A., Kralj, S.: Assembling of topological defects at neck-shaped membrane parts. Physica Status Solidi (a) **216**(13), 1800722 (2019)

[17] Käs, J., Sackmann, E.: Shape transitions and shape stability of giant phospholipid vesicles in pure water induced by area-to-volume changes. Biophysical Journal **60**(4), 825–844 (1991)

[18] Kralj-Iglič, V., Pocsfalvi, G., Mesarec, L., Šuštar, V., Hägerstrand, H., Iglič, A.: Minimizing isotropic and deviatoric membrane energy - an unifying formation mechanism of different cellular membrane nanovesicle types. PloS One **15**(12), 1–25 (2020)

[19] Deuling, H.J., Helfrich, W.: Red blood cell shapes as explained on the basis of curvature elasticity. Biophysical Journal **16**, 861–868 (1976)

[20] Mesarec, L., Iglič, A., Kralj-Iglič, V., Góźdź, W., Virga, E.G., Kralj, S.: Curvature potential unveiled topological defect attractors. Crystals **11**(5), 539 (2021)

[21] Kralj-Iglič, V., Heinrich, V., Svetina, S., Žekš, B.: Free energy of closed membrane with anisotropic inclusions. The European Physical Journal

B-Condensed Matter and Complex Systems **10**(1), 5–8 (1999)

[22] Deuling, H., Helfrich, W.: The curvature elasticity of fluid membranes: a catalogue of vesicle shapes. Journal de Physique **37**(11), 1335–1345 (1976)

[23] Iglič, A., Kralj-Iglič, V., Majhenc, J.: Cylindrical shapes of closed lipid bilayer structures correspond to an extreme area difference between the two monolayers of the bilayer. Journal of Biomechanics **32**(12), 1343–1347 (1999)

[24] Iglič, A., Kralj-Iglič, V., Božič, B., Bobrowska-Hägerstrand, M., Isomaa, B., Hägerstrand, H.: Torocyte shapes of red blood cell daughter vesicles. Bioelectrochemistry **52**(2), 203–211 (2000)
